# Detection of the Cell Cycle-Regulated Negative Feedback Phosphorylation of Mitogen-Activated Protein Kinases in Breast Carcinoma using Nanofluidic Proteomics

**DOI:** 10.1038/s41598-018-28335-8

**Published:** 2018-07-03

**Authors:** Yasuyo Urasaki, Ronald R. Fiscus, Thuc T. Le

**Affiliations:** 1 College of Pharmacy, Roseman University of Health Sciences, 10530 Discovery Drive, Las Vegas, NV 89135 USA; 2 College of Medicine, Roseman University of Health Sciences, 10530 Discovery Drive, Las Vegas, NV 89135 USA

## Abstract

Mitogen-activated protein kinases (MAPKs) play an important role in the regulation of cell proliferation, oncogenic transformation, and drug resistance. This study examined the capability of nanofluidic proteomics to identify aberrations in the MAPK signaling cascade, monitor its drug response, and guide the rational design of intervention strategies. Specifically, the protein post-translational modification (PTM) profiles of MEK1, MEK2, and ERK1/2 were measured in breast carcinoma and breast cancer cell lines. Nanofluidic proteomics revealed hyper-phosphorylation of MAPKs in breast carcinoma and breast cancer cells treated with kinase inhibitors that interfere with cell cycle regulation, such as dinaciclib, an inhibitor of cyclin-dependent kinases, and rigosertib, an inhibitor of polo-like kinase 1. A pMEK1 (Thr286) phosphor-isoform, which serves as a biomarker of cell cycle-regulated negative feedback phosphorylation in breast cancer cells, was detected in breast carcinoma. Inhibition of the MAPK pathway with dabrafenib, a B-Raf inhibitor, or trametinib, a MEK1/2 inhibitor, suppressed both the positively regulated phosphorylation of MAPKs and the negatively regulated phosphorylation of MEK1. Interestingly, the combinations of dabrafenib and rigosertib or trametinib and rigosertib permitted the suppression of positively regulated MAPK phosphorylation together with the promotion of negatively regulated MEK1 phosphorylation. The effectiveness of protein PTM-guided drug combinations for inhibition of the MAPK pathway remains to be experimentally tested. Via protein PTM profiling, nanofluidic proteomics provides a robust means to detect anomalies in the MAPK signaling cascade, monitor its drug response, and guide the possible design of drug combinations for MAPK pathway-focused targeting.

## Introduction

In the last several decades, cancer treatment has progressively evolved from non-specific cytotoxic chemotherapy toward selective mechanism-based therapeutics^1^. This therapeutic revolution is led by clinical success in cancer treatment via the use of small-molecule kinase inhibitors to target kinases whose mutations drive cancer growth and development^2^. The burgeoning library of molecular targeted drugs that interfere with specific oncogenic abnormalities ushers endless possibilities for cancer therapy^3,4^. However, the realization of molecular targeted cancer therapy is hindered by multiple challenges, such as the fact that only some human cancers have known kinase-domain mutations^5–8^ and the rapid development of drug resistance due to intrinsic inter- and intra-tumor heterogeneity^9,10^.

To overcome such challenges, molecular targeted cancer therapy is being applied more broadly, extending beyond specific oncogenic lesions to encompass aberrant signaling pathways whose components are not necessarily mutated^5^. Furthermore, multi-component therapy with combinations of molecular targeted drugs is being pursued to overcome drug resistance^11^. Past and current clinical trials for anti-cancer drug combinations have followed three broad categories that maximize the inhibition of a specific target by using multiple inhibitors against the same target, inhibition of a pathway by targeting multiple pathway components, or inhibition of multiple pathways representing multiple cellular processes^12^. However, these clinical trials have had limited success due to the lack of a rational drug combination strategy based on mechanisms of interaction between drugs. Currently, the enrollment of patients into clinical trials is not based on the sensitivity of an individual patient’s tumor to individual drugs or drug combinations^12^. A strong reliance on non-specific cytotoxicity for the phenotypic screening of anti-cancer drugs also hampers the evaluation of their molecular effects and the identification of biomarkers of drug sensitivity or resistance^13,14^. Future successes of multi-component anti-cancer therapy are dependent on the improvement of phenotypic screening methods to select cancer patients and evaluate drugs’ molecular effects^13,15,16^. In addition, non-clinical models for the rational design of drug combinations with predictive clinical outcomes are highly desired^12,15^.

A potential approach to cancer phenotypic screening is potentially found with nanofluidic proteomics, which can identify aberrant signaling pathways in cancer cells and monitor their responses to anti-cancer therapy. Previously, nanofluidic proteomics using capillary isoelectric focusing (cIEF) immunoassays has been used to detect aberrant signaling pathways in various diseases using nanograms of tissue biopsies^17–24^. Nanofluidic proteomics has also been deployed to detect oncoprotein activation in clinical specimens following treatment with anti-cancer drugs^22,25^. Nanofluidic proteomics has the potential to be a robust method that can identify cancer phenotypes, assist in the design of pathway-focused therapy, and screen for the molecular effects of individual drugs or drug combinations.

In this study, nanofluidic proteomics was deployed to monitor the signaling activity of the MAPK pathway in breast cancer cell lines and breast carcinoma biopsies. Specifically, the protein PTM profiles of MEK1, MEK1, ERK1/2 were measured. Changes in the protein PTM profiles as a function of drug treatment were measured to assess the drug effects on the MAPK pathway. The MAPK signaling cascade is a conserved pathway that regulates cellular proliferation, differentiation, survival, and migration^26^. Deregulation of the MAPK pathway is associated with many cancers in humans^6,27,28^. Targeting the MAPK pathway for anti-cancer therapeutics is being aggressively pursued with individual or combinations of small-molecule kinase inhibitors^8,28–30^. This study examined the capability of nanofluidic proteomics to identify aberrations in the MAPK pathway, monitor its drug response, and guide the rational design of drug combinations for MAPK pathway-focused targeting.

## Results

### Detection of protein phosphor-isoforms using nanofluidic proteomics

**Figure 1:**
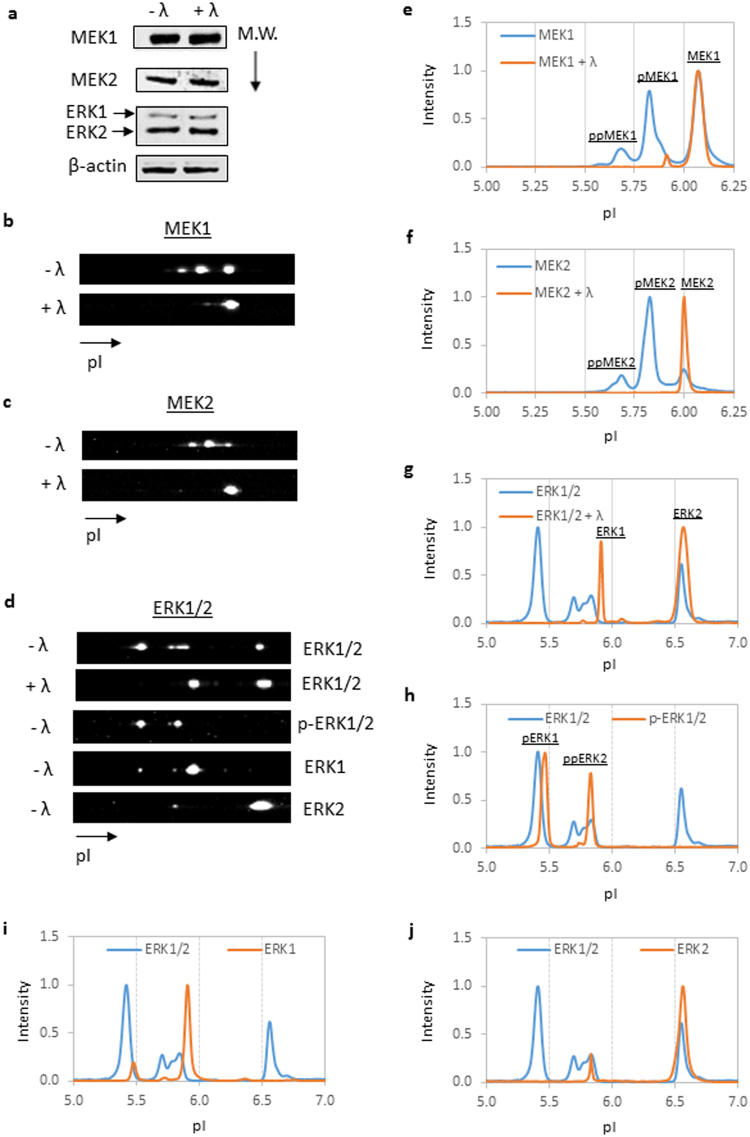
Identification of protein isoforms of the MAPK pathway using capillary isoelectric focusing (cIEF) immunoassays. (**a**) Western blotting with antibodies directed against MEK1, MEK2, and ERK1/2 proteins using total cell extracts (TCEs) of the breast cancer cell line BT474. TCEs of the BT474 cell line were either untreated (−λ) or treated (+λ) with λ phosphatase prior to Western blot assays. β-actin served as a loading control. Cropped Western blots were from different gels loaded with the same amount of TCEs. All gels were run on the same day and subjected to the same experimental procedures, including the same exposure duration during detection. A representative immunoblot of β-actin is presented to highlight comparable TCE loading between lanes. M.W.: molecular weight. (**b–d**) Raw cIEF immunoassays of (**b**) MEK1, (**c**) MEK2, and (**d**) ERK1/2 using TCEs of BT474 cell line without (−λ) or with (+λ) λ phosphatase treatment. Specific antibodies directed against both ERK1 and ERK2 isoforms (ERK1/2), the ERK1/2 phosphor-isoform (p-ERK1/2), the ERK1 isoform, or the ERK2 isoform were used to resolve ERK1/2 isoforms. Graphical presentations of cIEF immunoassays in **b–d** for (**e**) MEK1, (**f**) MEK2, (**g**) ERK1/2, (**h**) pERK1/2, (**i**) ERK1, and (**j**) ERK2. The peak intensities were normalized to 1 for all cIEF immunoassay data. pI: isoelectric point.

First, two methods of protein detection, Western blotting and cIEF immunoassay, were deployed to profile MEK1, MEK2, and ERK1/2 proteins in total cell extracts (TCEs) of a breast cancer cell line BT474. TCEs of BT474 were either untreated (−λ) or treated with λ phosphatase (+λ) to remove protein phosphor-isoforms. Western blots separated proteins based on their molecular weights, and changes in the protein migration patterns following λ phosphatase treatment were not detectable (Fig. 1a), a finding that was expected due to the very small change in protein molecular weight associated with a phosphorylation event. By contrast, cIEF immunoassays separated proteins based on charges and could detect changes in the protein migration patterns following λ phosphatase treatment (Fig. 1b–d). To distinguish ERK1 and ERK2 from their phosphor-isoforms, primary antibodies specific to p-ERK1/2, ERK1, and ERK2 were used for analysis (Fig. 1d, bottom three panels). Alternatively, the cIEF immunoassay data were presented graphically as a function of the isoelectric points versus intensity (Fig. 1e–j). The isoelectric points corresponding to the protein isoforms are summarized in Table 1. The pI range assignments for protein isoforms were experimentally determined by this study as well as by several previous studies from multiple independent research groups^17–20,23,31,32^. Compared with size separation by Western blots, charge separation by the cIEF immunoassay was highly sensitive to the analysis of protein isoforms of the MAPK pathway.

**Table 1:** Assignment of pI ranges to protein isoforms.

Protein Isoform	pI Range
MEK1	6.00–6.25
pMEK1	5.75–6.00
ppMEK1	5.60–5.75
pppMEK1	5.40–5.60
ppppMEK1	5.20–5.40
MEK2	5.90–6.10
pMEK2	5.75–5.90
ppMEK2	5.60–5.75
ERK1	5.90–6.00
pERK1	5.30–5.50
ppERK1	5.00–5.30
ERK2	6.40–6.60
pERK2	6.10–6.40
ppERK2	5.50–5.90

### Profiling protein isoforms of the MAPK pathway in breast cancer and non-cancerous cell lines

**Figure 2:**
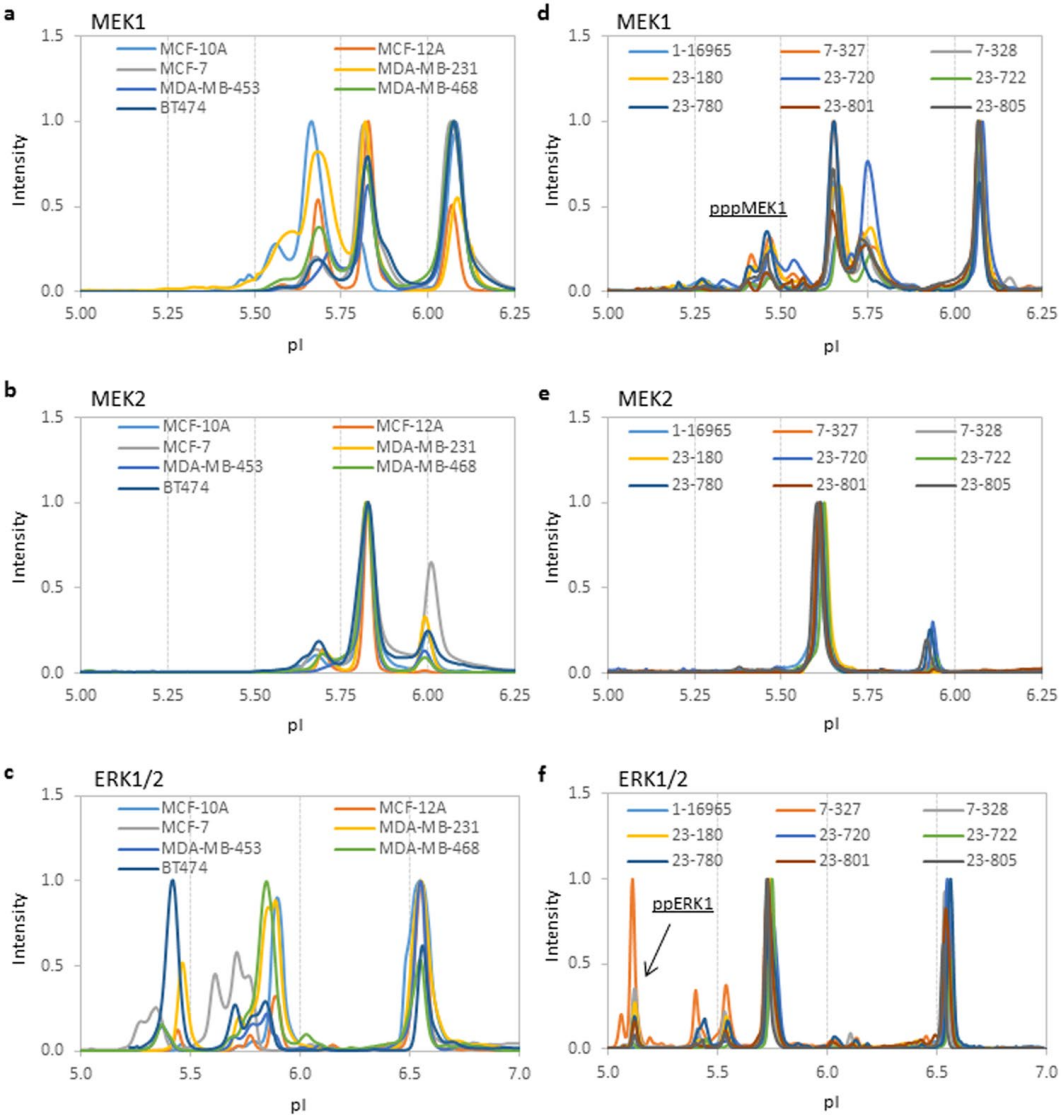
Distribution of protein isoforms of the MAPK pathway in breast cancer cell lines and breast carcinoma. (**a**–**c**) cIEF immunoassay profiles of (**a**) MEK1, (**b**) MEK2, and (**c**) ERK1/2 in 2 non-tumorigenic epithelial cell lines, MCF-10A and MCF-12A, and 5 breast cancer cell lines, MCF-7, MDA-MB-231, MDA-MB-453, MDA-MB-468, and BT474. (**d**–**f**) cIEF immunoassay profiles of (**d**) MEK1, (**e**) MEK2, and (**f**) ERK1/2 in 9 breast carcinoma samples. The peak intensities were normalized to 1 for all cIEF immunoassay data.

Next, cIEF immunoassays were deployed to measure the protein isoform profiles of MEK1, MEK2, and ERK1/2 in seven cell lines (Table 2). These cell lines included five breast cancer cell lines (MCF-7, MDA-MB-231, MDA-MB-453, MDA-MB-468, and BT474) and two non-tumorigenic epithelial cell lines of breast tissues (MCF-10A and MCF-12A). Although there was significant variability among the cell lines, MEK1 isoforms were generally distributed among three major populations, MEK1, pMEK1, and ppMEK1 (Fig. 2a). Similarly, MEK2 isoforms were distributed among three major populations, MEK2, pMEK2, and ppMEK2, with pMEK2 being the dominant isoform in all cell lines (Fig. 2b). Expectedly, ERK1 and ERK2 and their phosphor-isoforms pERK1 and ppERK2 were present in all cell lines (Fig. 2c).

**Table 2:** Human breast cancer and non-cancerous cell lines.

Cell line	Subtype	ER	PR	HER2	Source	Tumor type	Age	Ethnicity
MCF-10A	Basal B	−	−	−	P. Br	F, NT	36	W
MCF-12A	Basal B	−	−	−	P. Br	F, NT	60	W
MCF-7	Luminal A	+	+	−	PE	IDC	69	W
MDA-MB-231	Claudin-low	−	−	−	PE	AC	51	W
MDA-MB-453	HER2	−	−	+	PF	AC	48	W
MDA-MB-468	Basal A	−	−	−	PE	AC	51	B
BT474	Luminal B	+	+	+	P.Br	IDC	60	W

ER: estrogen receptor; PR: progesterone receptor; HER2: human epidermal growth factor 2; P. Br: primary breast; PE: pleural effusion; F: fibrocystic disease; NT: non-tumorous; IDC: invasive ductal carcinoma; AC: adenocarcinoma; W: white; B: black.

### Profiling protein isoforms of the MAPK pathway in breast carcinoma

Using tissue biopsies from nine patients with breast carcinoma (Table 3), the protein isoform profiles of MEK1, MEK2, and ERK1/2 were measured using cIEF immunoassays. Interestingly, the protein PTM profiles of the MAPK pathway in breast carcinoma were significantly elevated compared with those in cell lines. In addition to the three MEK1 isoforms of MEK1, pMEK1, and ppMEK1 observed in cell lines, the pppMEK1 isoform was clearly observable in all breast carcinomas (Fig. 2d). On the other hand, MEK2 isoforms were present in two populations of MEK2 and ppMEK2, with ppMEK2 having a prevalence greater than 90% (Fig. 2e). Moreover, the presence of ppERK1, which was absent in the breast cancer cell lines, was detected in breast carcinoma (Fig. 2f). While the ERK2 and ppERK2 isoforms were evenly distributed, only pERK1 and ppERK1, but not ERK1, were observed.

**Table 3:** Human primary breast carcinoma.

Case ID	Gender	Age	Ethnicity	Pathology Diagnosis	Stage	Grade
1–16965	Female	38	Caucasian	No data	IIB	No data
7–327	Female	62	Caucasian	Infiltrating lobular carcinoma	IIB	G3
7–328	Female	35	Caucasian	Intraductal carcinoma	IA	G1
23–180	Female	47	Caucasian	Infiltrating ductal carcinoma	IIA	G2
23–720	Female	56	Caucasian	Infiltrating ductal carcinoma	III	G2
23–722	Female	55	Caucasian	Infiltrating ductal carcinoma	III	G2
23–780	Female	64	Caucasian	Infiltrating ductal carcinoma	III	G2
23–801	Female	61	Caucasian	Infiltrating ductal and lobular carcinoma	III	G2
23–805	Female	45	Caucasian	Infiltrating ductal carcinoma	IV	No data

### Inhibition of the MAPK pathway using small-molecule kinase inhibitors

**Figure 3:**
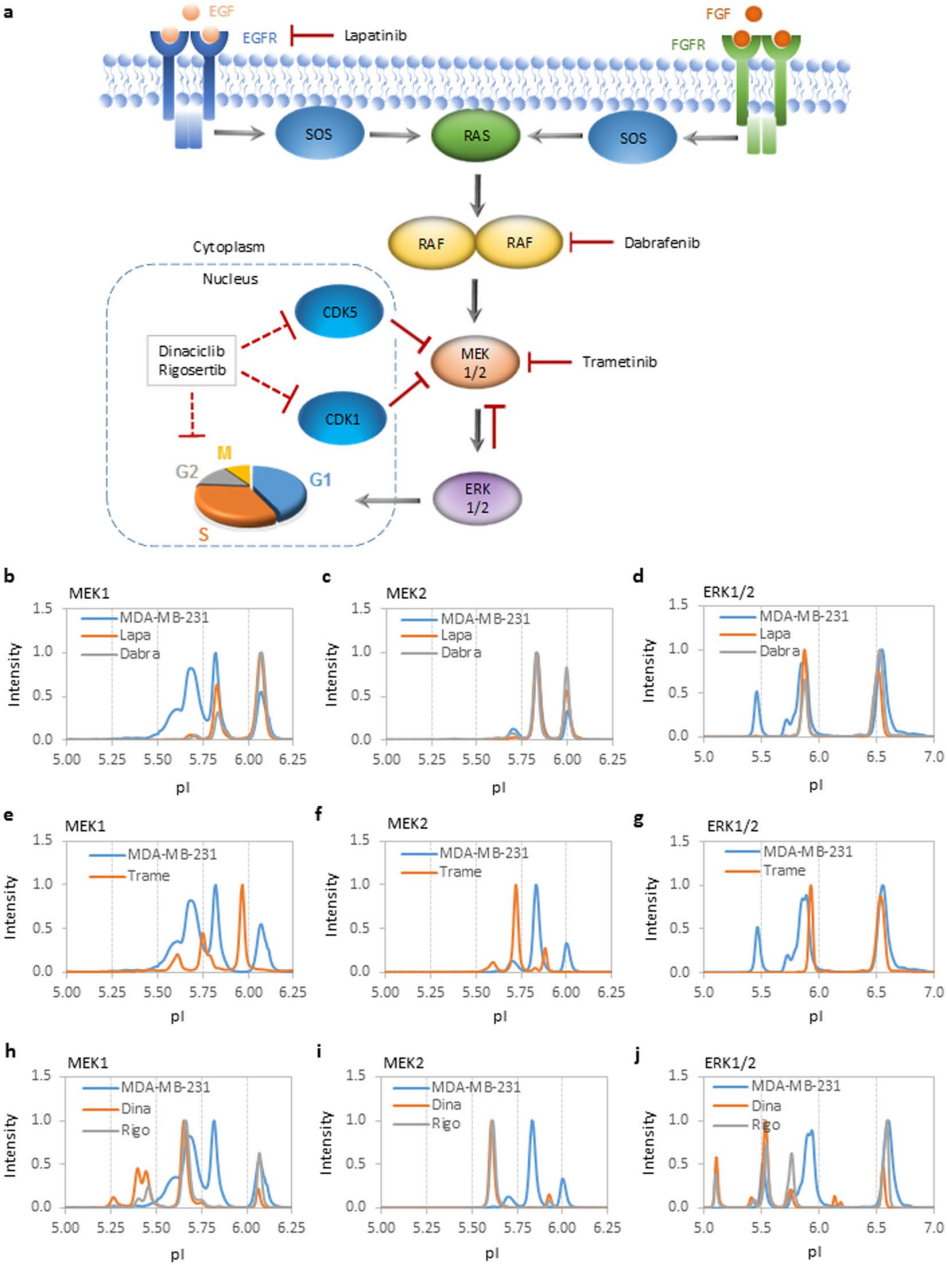
MAPK pathway and cell cycle inhibition cause perturbations in the distribution of protein isoforms. (**a**) Diagram of the MAPK signaling pathway and targets of small-molecule kinase inhibitors or negative feedback inhibition. EGF: epidermal growth factor; EGFR: epidermal growth factor receptor; FGF: fibroblast growth factor; FGFR: fibroblast growth factor receptor; SOS: a guanine nucleotide exchange factor; RAS: a small GTPase; RAF: a serine/threonine-specific protein kinase; MEK: mitogen-activated protein kinase kinase; ERK: mitogen-activated protein kinase; CDK: cyclin-dependent kinase; solid red blunted arrows: direct inhibition; dashed red blunted arrows: direct or indirect inhibition; gray arrows: direction of signaling cascade. (**b**) Lapatinib and dabrafenib reduced the phosphorylation of MEK1, MEK2, and ERK1/2. (**c**) Trametinib caused shifts to lower pI values for MEK1 and MEK2 and suppressed ERK1/2 phosphorylation. (**d**) Dinaciclib and rigosertib increased the phosphorylation of MEK1, MEK2, and ERK1/2. All experiments were performed in the MDA-MB-231 breast cancer cell line. The peak intensities were normalized to 1 for all cIEF immunoassay data.

To shed light on possible sources of perturbation in the MAPK signaling activity in breast tumors, the protein phosphor-isoform profiles of MEK1, MEK2, and ERK1/2 were measured in MDA-MB-231 breast cancer cell lines treated with small-molecule kinase inhibitors. The small-molecule kinase inhibitors included lapatinib, which inhibits epidermal growth factor receptor^33^, dabrafenib, which inhibits B-Raf^34^, trametinib, which inhibits MEK1 and MEK2^35^, dinaciclib, which inhibits CDK1 and CDK5^36^, and rigosertib, which inhibits polo-like kinase 1^37^ (Fig. 3a). Lapatinib, dabrafenib, and trametinib interfere with positive regulation of the MAPK pathway. By contrast, dinaciclib and rigosertib cause cell cycle arrest and induce negative feedback regulation of the MAPK pathway^38,39^.

The small-molecule kinase inhibitors induced perturbations in MAPK signaling activity that could be characterized by three distinctive groups. The first group included lapatinib and dabrafenib, which suppressed phosphor-isoforms and promoted the unphosphorylated isoforms of MEK1, MEK2, and ERK1/2 (Fig. 3b–d). The second group included trametinib, which caused shifts toward lower pI values for MEK1 and MEK2 isoforms and suppressed phosphor-isoforms of ERK1/2 (Fig. 3e–g). It is important to note that shifts toward lower pI values for MEK1 and MEK2 induced by trametinib were resistant to λ phosphatase treatment (Supplemental Fig. S1a,b), suggesting that these shifts were not due to increased phosphorylation. It is plausible that the changes in the surface charges of MEK1 and MEK2 could be consequences of conformational changes in protein structures due to direct binding to trametinib. Finally, the third group included dinaciclib and rigosertib, which promoted the phosphorylation of MEK1, MEK2 and ERK1/2 and induced the appearance of pppMEK1, ppMEK2, and ppERK1 (Fig. 3h–j). Interestingly, the protein phosphorylation profiles of the MAPK pathway induced by dinaciclib and rigosertib in MDA-MB-231 cells resembled those of breast carcinoma.

### Positive and negative regulation of MEK1 with site-specific phosphorylation

**Figure 4:**
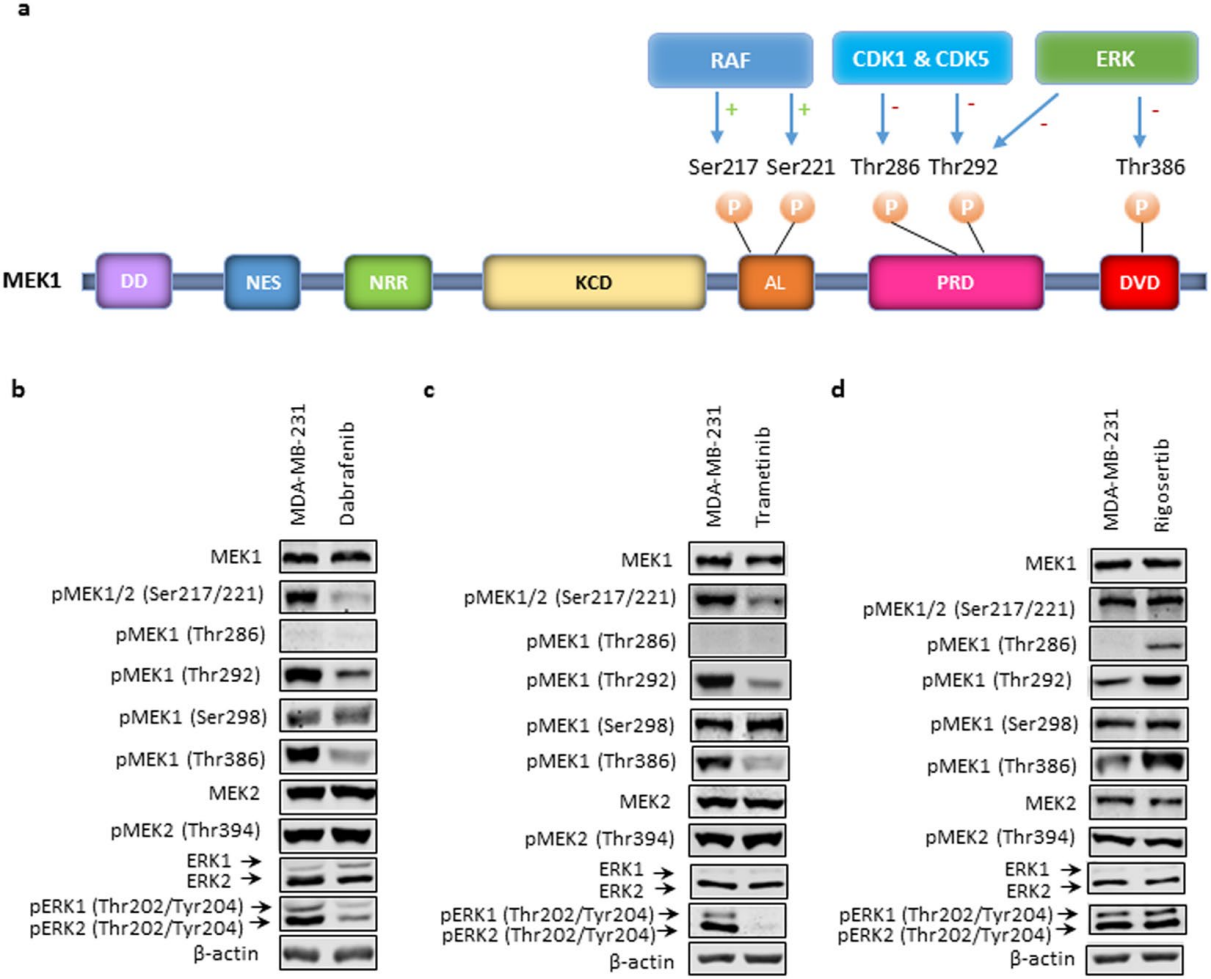
Positive and negative regulation of MEK1 by site-specific phosphorylation. (**a**) Linear representation of MEK1 protein and sites of phosphorylation. Phosphorylation at Ser217 and Ser221 residues by RAF positively regulates MEK1 activity. By contrast, phosphorylation at Thr286 and Thr292 by CDK1 and CDK5 and phosphorylation at Thr292 and Thr386 by ERK1/2 negatively regulates MEK1 activity. DD: docking domain; NES: nuclear export sequence; NRR: negative regulatory region; KCD: kinase catalytic domain; AL: activation loop; PRD: proline-rich domain; DVD: domain of versatile docking. (**b**–**d**) Western blot analyses of the expression and phosphorylation levels of MEK1, MEK2, and ERK1/2 in the MDA-MB-231 cell line without (left lane) and with (right lane) treatment with (**b**) dabrafenib, (**c**) trametinib, and (**d**) rigosertib. β-actin served as a loading control. Cropped Western blots were from different gels loaded with the same amount of TCEs. All gels were run on the same day and subjected to the same experimental procedures, including the same exposure duration during detection. Representative immunoblots of β-actin are presented to highlight comparable TCE loading between lanes.

MEK1 enzymatic activity is regulated by site-specific phosphorylation that can be activated with phosphorylation of Ser217/Ser221 by Raf kinase^40^ or suppressed by phosphorylation of Thr286 and Thr292 by CDK1 and CDK5^38,39,41^ or Thr292 and Thr386 by ERK1/2^42–44^ (Fig. 4a). To further examine the perturbations in MAPK signaling activity induced by small-molecule kinase inhibitors, Western blotting was performed using antibodies that recognize specific protein phosphor-isoforms (Fig. 4b–d). The Western blot data are summarized in Table 4. Briefly, dabrafenib or trametinib treatment suppressed pMEK1/2 (Ser217/Ser221), pMEK1(Thr292), pMEK1 (Thr386), and pERK1/2 (Thr202/Tyr204). On the other hand, rigosertib treatment increased pMEK1 (Thr286), pMEK1 (Thr292), and pMEK1 (Thr386) but had no effect on pMEK1/2 (Ser217/Ser221) or pERK1/2 (Thr202/Tyr204). Distinct regulatory mechanisms by small-molecule kinase inhibitors were observed with dabrafenib or trametinib treatment, which suppressed both the positive and negative regulation of MEK1, whereas rigosertib treatment promoted the negative regulation of MEK1.

**Table 4:** Summary of Western blot data of protein phosphor-isoforms following drug treatment

Phosphor-isoform	Dabrafenib	Trametinib	Rigosertib	Dabra & Rigo	Trame & Rigo
pMEK1/2 (Ser217/Ser221)	−	−	NC	−	NC
pMEK1 (Thr286)	ND	ND	+	+	ND
pMEK1 (Thr292)	−	−	+	NC	NC
pMEK1 (Ser298)	NC	NC	NC	NC	NC
pMEK1 (Thr386)	−	−	+	NC	NC
pMEK2 (Thr394)	NC	NC	NC	NC	NC
pERK1/2 (Thr202/Tyr204)	−	−	NC	−	−

ND: not detectable; NC: no change; −: decreased; + : increased.

### Measuring the effects of drug combinations on the distribution of MAPK pathway protein isoforms

**Figure 5:**
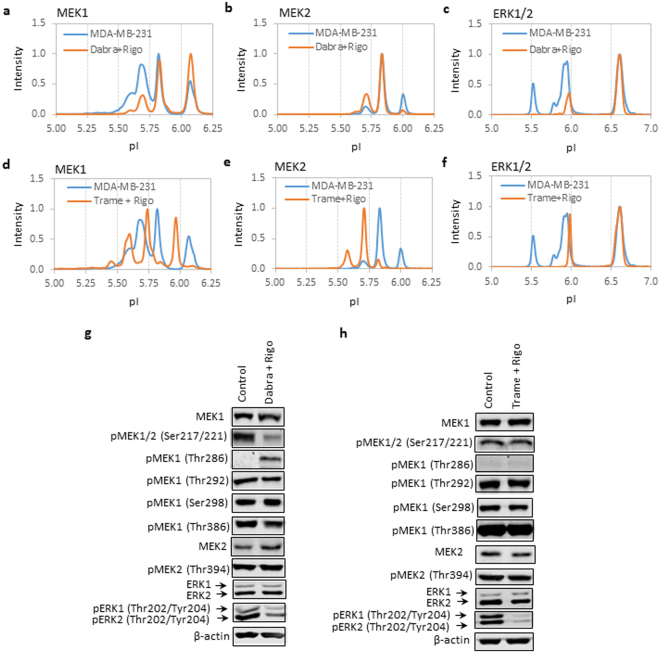
Effects of drug combinations on protein phosphorylation of the MAPK pathway. (**a**–**c**) cIEF immunoassay profiles of (**a**) MEK1, (**b**) MEK2, and (**c**) ERK1/2 in the MDA-MB-231 cell line without (blue line) or with (orange line) treatment with both dabrafenib and rigosertib. (**d**–**f**) cIEF immunoassay profiles of (**d**) MEK1, € MEK2, and (**f**) ERK1/2 in MDA-MB-231 cells without (blue line) or with (orange line) treatment with a combination of trametinib and rigosertib. Peak intensities were normalized to 1 for all cIEF immunoassay data. (**g**,**h**) Western blot analyses of the expression and phosphorylation levels of MEK1, MEK2, and ERK1/2 in MDA-MB-231 cells without (left lane) and with (right lane) treatment with a (**g**) combination of dabrafenib and rigosertib or (**h**) a combination of trametinib and rigosertib. β-actin served as a loading control. Cropped Western blots were from different gels loaded with the same amount of TCEs. All gels were run on the same day and subjected to the same experimental procedures, including the same exposure duration during detection. Representative immunoblots of β-actin are presented to highlight comparable TCE loading between lanes.

To understand how distinct regulatory mechanisms interact, MDA-MB-231 cells were treated with combinations of dabrafenib and rigosertib or trametinib and rigosertib. Both cIEF immunoassays and Western blotting were performed to measure the protein phosphor-isoform profiles of the MAPK pathway. cIEF immunoassays revealed that treatment with a combination of dabrafenib and rigosertib reduced the ppMEK1 isoform, increased the ppMEK2 isoform, and suppressed the ERK1/2 phosphor-isoforms (Fig. 5a–c). Treatment with a combination of trametinib and rigosertib caused shifts to lower pI values for both MEK1 and MEK2 and suppressed ERK1/2 phosphor-isoforms (Fig. 5d–f). On the other hand, Western blotting revealed that treatment with a combination of dabrafenib and rigosertib suppressed pMEK1/2 (Ser217/Ser221) and pERK1/2 (Thr202/Tyr204) phosphor-isoforms and increased pMEK1 (Thr286) (Fig. 5g). The combination of dabrafenib and rigosertib appeared to suppress the positive regulation and promote the negative regulation of MEK1 (Fig. 5h). Treatment with a combination of trametinib and rigosertib caused only suppression of the pERK1/2 (Thr202/Tyr204) phosphor-isoform but had no observable effect on the MEK1 or MEK2 phosphor-isoforms.

### The cell cycle-regulated negative feedback phosphorylation of MEK1 in breast carcinoma

**Figure 6:**
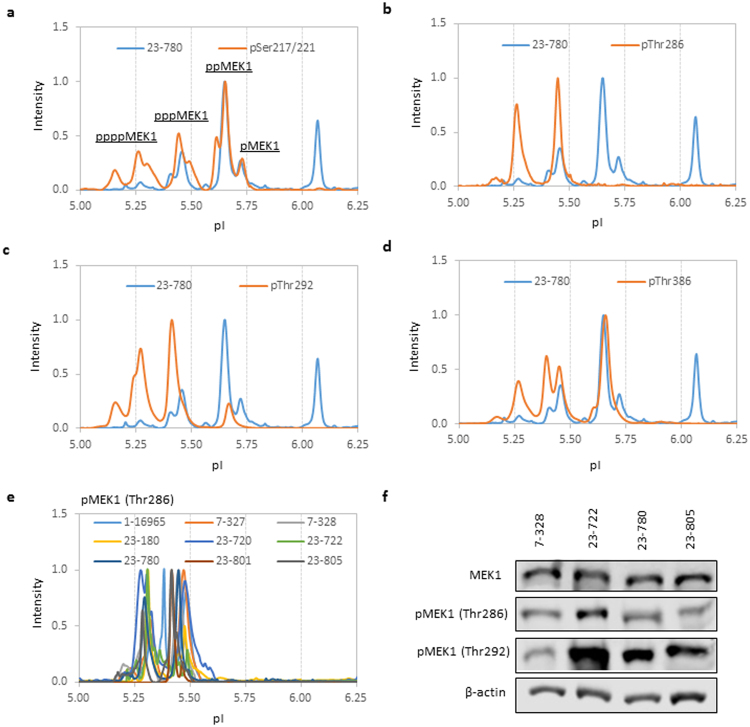
Evidence of the cell cycle-regulated feedback phosphorylation of MEK1 in breast carcinoma. (**a**–**d**) Detection of specific MEK1 phosphor-isoforms in breast carcinoma ID no. 23–780 probed with primary antibodies specific for (**a**) pSer217/221, (**b**) pThr286, (**c**) pThr292, and (**d**) pThr386. (**e**) Detection of pMEK1 (Thr286) phosphor-isoforms in all nine breast carcinoma samples. (**f**) Detection of pMEK1 (Thr286) and pMEK1 (Thr292) phosphor-isoforms in selected breast carcinoma samples by Western blotting. β-actin served as a loading control. Cropped Western blots were from different gels loaded with the same amount of TCEs. All gels were run on the same day and subjected to the same experimental procedures, including the same exposure duration during detection. A representative immunoblot of β-actin is presented to highlight comparable TCE loading between lanes.

cIEF immunoassays and Western blotting were performed to identify specific MEK1 phosphor-isoforms that contribute to the presence of pppMEK1 in selected breast carcinoma samples. Using primary antibodies that recognize MEK1 phosphor-isoforms at pSer217/221, pThr286, pThr292, and pThr386, cIEF immunoassays were performed in selected breast carcinoma samples. A representative set of cIEF immunoassay data is presented in Fig. 6a–d. Briefly, four MEK1 phosphor-isoforms from pMEK1 to ppppMEK1 were detected with a primary antibody specific to pSer217/221 (Fig. 6a). Two MEK1 phosphor-isoforms, pppMEK1 and ppppMEK1, were detected with a primary antibody specific to pThr286 (Fig. 6b). Three MEK1 phosphor-isoforms, ppMEK1, pppMEK1, and ppppMEK1, were detected with a primary antibody specific to pThr292, where the ppMEK1 population was less than 10% of all phosphor-isoforms (Fig. 6c). Three MEK1 phosphor-isoforms, ppMEK1, pppMEK1, and pppMEK1, were detected with a primary antibody specific to pThr386, where the ppMEK1 population comprised up to 40% of all the phosphor-isoforms (Fig. 6d). cIEF immunoassay data indicated that MEK1 phosphor-isoforms at pThr286 and pThr292 were present as pppMEK1 and ppppMEK1. Indeed, analyses of all nine breast carcinoma samples with a primary antibody specific to pThr286 revealed the exclusive presence of pThr286 as pppMEK1 and ppppMEK1 isoforms. Furthermore, Western blotting was performed using primary antibodies specific to pThr286 and pThr292 in four selective breast carcinoma samples. Breast carcinoma samples were selected based on the sufficiency of TCEs for Western blot analyses. Consistent with the cIEF immunoassay data, MEK1 phosphor-isoforms at pThr286 and pThr292 were detected (Fig. 6d).

## Discussion

Using cIEF immunoassays, this study reported hyper-phosphorylation of the MAPK pathway in breast carcinoma compared with that in breast cancer cell lines. Specifically, the triple-phosphorylated pppMEK1 isoform and double-phosphorylated ppERK1 isoform were present in breast carcinoma but absent in breast cancer cell lines. In addition, double-phosphorylated ppMEK2 was the dominant isoform in breast carcinoma, whereas mono-phosphorylated pMEK2 was the dominant isoform in breast cancer cell lines. Interestingly, treatment with small-molecule kinase inhibitors that cause cell cycle arrest, such as rigosertib and dinaciclib, promoted hyper-phosphorylation of the MAPK pathway in breast cancer cell lines. Western blot analyses revealed that the hyper-phosphorylation of MEK1 induced by rigosertib treatment was due to increased phosphorylation at negative regulatory sites at amino acid residues Thr286, Thr292, and Thr386. Both cIEF immunoassays and Western blotting confirmed the presence of the pMEK1 (Thr286) phosphor-isoform in selected breast carcinoma samples, indicating cell cycle-regulated feedback phosphorylation. Furthermore, cIEF immunoassays revealed that the pMEK1 (Thr286) phosphor-isoform contributed to the presence of the pppMEK1 isoform in all breast carcinoma samples examined.

This study revealed that the protein PTM profile could be used to identify aberrations in a signaling pathway and assist in the design of pathway-focused therapy. For example, the hyper-phosphorylation of MEK1, MEK2, and ERK1/2 in breast carcinoma samples indicated hyper-activity of the MAPK pathway. In addition, the presence of the pMEK1 (Thr286) phosphor-isoform in breast carcinoma indicated negative feedback phosphorylation that could stem from cell cycle dysregulation. When breast cancer cells were treated with dabrafenib or trametinib, both the positively regulated phosphorylation at Ser217/221 and negatively regulated phosphorylation at Thr292 and Thr386 of MEK1 were suppressed. This was likely a consequence of the reduced positively regulated phosphorylation of ERK1/2 at Thr202/Tyr204, leading to inhibition of the negative feedback phosphorylation of MEK1 at Thr292 and Thr386 by ERK1/2. By contrast, treatment of breast cancer cells with rigosertib promoted the negative feedback phosphorylation of MEK1 at Thr286, Thr292, and Thr386 without affecting the positively regulated phosphorylation of MEK1 at Ser217/221 or ERK1/2 at Thr202/Tyr204. Theoretically, inhibition of a pathway could be achieved most effectively by the suppression of positive regulation coupled with the promotion of negative regulation. Treatment of breast cancer cells with the combinations of both dabrafenib and rigosertib and trametinib and rigosertib achieved this design objective. The combination of dabrafenib and rigosertib suppressed the positively regulated phosphorylation of MEK1 at Ser217/221 and ERK1/2 at Thr202/Tyr204, promoted the negatively regulated phosphorylation of MEK1 at Thr286, and maintained the negatively regulated phosphorylation of MEK1 at Thr292 and Thr386. On the other hand, treatment of breast cancer cells with a combination of trametinib and rigosertib suppressed the positively regulated phosphorylation of ERK1/2 at Thr202/Tyr204 and maintained the negatively regulated phosphorylation of MEK1 at Thr292 and Thr386. Future studies will experimentally investigate the effectiveness of drug combinations guided by the protein PTM profiles of a specific signaling protein for pathway-focused targeting.

Consistent with many previous studies, cIEF immunoassays were highly suitable for the analysis of protein PTM profiles in finite tissue biopsies^45^. Typically, only 40 ng of TCE were used per cIEF immunoassay^46^. By comparison, a 1D Western blot required approximately 10 µg for molecular weight analysis, and 2D Western blotting required approximately 500 µg for both charges and molecular weight analyses^32^. cIEF immunoassays were highly sensitive to the detection of various modes of protein PTM, including phosphorylation, acetylation, and O-linked glycosylation^17,21,47–50^. This capability allows cIEF immunoassays to detect changes in the signaling or enzymatic activities of a protein in diseased states or in response to drug treatment. A drawback of the cIEF immunoassay is its inability to identify the specific sites of PTM that provide critical information on the regulation of protein activity. This deficiency is somewhat mitigated by the use of selective antibodies that recognize the specific phosphorylation sites of a protein. However, the availability of these selective antibodies is highly limited. Due to the lack of antibodies that recognize specific phosphorylation sites, which amino acid residues on ppMEK2 or ppERK1 received phosphorylation in breast carcinoma or breast cancer cells following treatment with small-molecule kinase inhibitors that target cell cycle regulation remain unclear. An emerging proteomic technology that couples cIEF with mass spectrometry has the potential to overcome the current limitation of cIEF immunoassays and advance protein PTM profiling capability^51^.

The MAPK signaling cascade plays an important role in the regulation of cell proliferation, oncogenic transformation, and drug resistance^52,53^. Several small-molecule kinase inhibitors that target components of the MAPK signaling cascade have been approved by the US Food and Drug Administration for the treatment of *BRAF*-mutant melanoma, including trametinib, dabrafenib, and vemurafenib, or the treatment of kidney and liver cancers with sorafenib^1^. However, the efficacy of these kinase inhibitors on other tumors have been somewhat limited^28,54^. Numerous clinical trials are ongoing using combinations of MEK1/2 inhibitors with BRAF inhibitors for the same pathway inhibition or MEK1/2 inhibitors with PI3K inhibitors for dual pathway inhibition^28,30,54^. Nonetheless, there is currently a lack of predictive biomarkers of sensitivity or resistance to kinase inhibitors, consequently hindering the development of a pre-clinical model that can identify the best combinations and predict clinical success^12,13,15,28,30,54^. Nanofluidic proteomics could complement other emerging technology platforms^55–57^ and model systems^16^ that seek to identify drug resistance biomarkers and design effective drug combinations that target MAPK or other oncogenic signaling pathways for personalized anti-cancer therapy.

## Methods

### Cell lines and drug treatment

All cell lines were obtained from American Type Culture Collection (ATCC, Manassas, VA) and cultured according to a published guideline^58,59^. All small-molecule kinase inhibitors were obtained from Selleckchem (Houston, TX). Cells were treated with kinase inhibitors for 24 hours prior to collection and analysis. The concentrations of kinase inhibitors included 5 µM lapatinib, 10 µM dabrafenib, 0.5 µM trametinib, 0.1 µM dinaciclib, and 1 µM rigosertib, which were chosen based on the EC_50_ values determined by our own cytotoxic screening assays.

### Human primary breast carcinoma biopsies

De-identified fresh-frozen biopsies from nine patients with breast carcinoma (~100–200 mg/biopsy) were acquired from Cureline, Inc. (Brisbane, CA) and maintained in liquid nitrogen until usage. This research involved only the study of existing pathological specimens that were publicly available and eligible for exemption under 45 CFR 46.101(b)(4) from 45 CFR part 46 requirements by the Office for Human Research Protections at the U.S. Department of Health and Human Services.

### Preparation of TCEs

Cultured cells (~10^6^ cells) or breast carcinoma biopsies (~50 mg) were added to Bicine/CHAPS Lysis Buffer (cat. no. 040-764, Protein Simple, Santa Clara, CA; 60 µl for cultured cells and 250 µl for breast carcinoma biopsies) containing proteinase and phosphatase inhibitors and were homogenized twice for 6 seconds. Homogenates were incubated on ice for 10 minutes, sonicated 4 times for 5 seconds each, rotated at 4 °C for 2 hours, and centrifuged at 12000 rpm in an Eppendorf 5430R microfuge for 20 minutes at 4 °C. Supernatants were collected as TCEs for both Western blotting and cIEF immunoassays. For Western blotting, the protein concentrations of TCEs were estimated using the CB-X protein assay kit (cat. no. 786-12, G-Biosciences, St. Louis, MO), adjusted with 4× SDS sample buffer containing 2-mercaptoethanol, boiled for 5 minutes and used for analysis. For cIEF immunoassays, the TCEs were prepared in a Premix G2 pH 5–8 separation gradient containing pI standards (Protein Simple) and added to 384-well plates for analysis.

### Treatment with λ phosphatase

Approximately 1 µl of 𝜆 phosphatase (cat. no. 14–405; Merck Millipore, Billerica, MA) was added to 1 µl of reaction buffer (final concentrations of 5 mM DDT, 50 mM Hepes, 100 µM EDTA, 2 mM MnCl_2_) and 8 µl of TCEs (2 mg/ml). The mixture was incubated at 37 °C for 30 minutes, chilled on ice to stop the reaction, and used for Western blotting or cIEF immunoassays.

### Western blotting

TCEs were separated on 10% SDS-PAGE gels, transferred to nitrocellulose membranes, incubated first with the primary antibodies against proteins or protein phosphor-isoforms of interest (Supplemental Table S1) and then with IRDye 680RD secondary antibodies (cat. no. 92668070, LI-COR, Lincoln, NE). Immunoblots were detected using the LI-COR’s Odyssey CLx imaging system. Membranes were stripped and re-incubated with antibodies against β-actin, which served as a loading control.

### cIEF immunoassays

cIEF immunoassays were performed using the NanoPro 1000 system (Protein Simple). Samples of 400-nanoliter volume were separated by isoelectric focusing using the 96-capillary system, followed by immobilization of the proteins onto the inner capillary walls with ultraviolet irradiation. Primary antibodies (Supplemental Table S1) and horseradish peroxidase-conjugated secondary antibodies (cat. no. 7074, Cell Signaling, Danvers, MA) were sequentially introduced into the capillaries, followed by chemiluminescence detection reagents. The incubation times were 110 and 55 minutes for the primary and secondary antibodies, respectively. The separation time was 50 minutes at 15,000 microwatts. On average, 40 ng of TCE was loaded into each capillary, and the standard exposure time during signal detection was 240 seconds. All the cIEF immunoassays were performed with a minimum of four repeats. High fidelity between repeated measurements was consistent with published reports with coefficient of variation values ≤ 0.1^45,46^.

### Data availability

The authors declare that data supporting the findings of this study are available within the paper and its supplementary information files.

## Electronic supplementary material

Supplementary Information

## References

[1] Gharwan H, Groninger H (2016). Kinase inhibitors and monoclonal antibodies in oncology: clinical implications. Nat. Rev. Clin. Oncol..

[2] Zhang J, Yang PL, Gray NS (2009). Targeting cancer with small molecule kinase inhibitors. Nat. Rev. Cancer.

[3] Wu P, Nielsen TE, Clausen MH (2015). FDA-approved small-molecule kinase inhibitors. Trends Pharmacol. Sci..

[4] Vanneman M, Dranoff G (2012). Combining immunotherapy and targeted therapies in cancer treatment. Nat. Rev. Cancer.

[5] Sawyers C (2004). Targeted cancer therapy. Nature.

[6] Dhillon AS, Hagan S, Rath O, Kolch W (2007). MAP kinase signalling pathways in cancer. Oncogene.

[7] Downward J (2003). Targeting RAS signalling pathways in cancer therapy. Nat. Rev. Cancer.

[8] Sebolt-Leopold JS, Herrera R (2004). Targeting the mitogen-activated protein kinase cascade to treat cancer. Nat. Rev. Cancer.

[9] Marusyk A, Almendro V, Polyak K (2012). Intra-tumour heterogeneity: a looking glass for cancer?. Nat. Rev. Cancer.

[10] Easwaran H, Tsai HC, Baylin SB (2014). Cancer epigenetics: tumor heterogeneity, plasticity of stem-like states, and drug resistance. Mol. Cell.

[11] Gross S, Rahal R, Stransky N, Lengauer C, Hoeflich KP (2015). Targeting cancer with kinase inhibitors. J. Clin. Invest..

[12] Dancey JE, Chen HX (2006). Strategies for optimizing combinations of molecularly targeted anticancer agents. Nat. Rev. Drug Discov..

[13] Moffat JG, Rudolph J, Bailey D (2014). Phenotypic screening in cancer drug discovery - past, present and future. Nat. Rev. Drug Discov..

[14] Kepp O, Galluzzi L, Lipinski M, Yuan J, Kroemer G (2011). Cell death assays for drug discovery. Nat. Rev. Drug Discov..

[15] Huang M, Shen A, Ding J, Geng M (2014). Molecularly targeted cancer therapy: some lessons from the past decade. Trends Pharmacol. Sci..

[16] Crystal AS (2014). Patient-derived models of acquired resistance can identify effective drug combinations for cancer. Science.

[17] Urasaki Y, Fiscus RR, Le TT (2016). Molecular classification of fatty liver by high-throughput profiling of protein post-translational modifications. J. Pathol..

[18] Padhan N (2016). High sensitivity isoelectric focusing to establish a signaling biomarker for the diagnosis of human colorectal cancer. BMC Cancer.

[19] Crosbie PA (2016). ERK and AKT phosphorylation status in lung cancer and emphysema using nanocapillary isoelectric focusing. BMJ Open Respir. Res..

[20] Padhan N (2017). Highly sensitive and specific protein detection via combined capillary isoelectric focusing and proximity ligation. Sci. Rep..

[21] Urasaki Y, Zhang C, Cheng JX, Le TT (2018). Quantitative assessment of liver steatosis and affected pathways with molecular imaging and proteomic profiling. Sci. Rep..

[22] Sabnis H, Bradley HL, Bunting ST, Cooper TM, Bunting KD (2014). Capillary nano-immunoassay for Akt 1/2/3 and 4EBP1 phosphorylation in acute myeloid leukemia. J. Transl. Med..

[23] Chen JQ (2013). Capillary isoelectric-focusing immunoassays to study dynamic oncoprotein phosphorylation and drug response to targeted therapies in non-small cell lung cancer. Mol. Cancer Ther..

[24] Tikhanovich I (2014). Regulation of FOXO3 by phosphorylation and methylation in hepatitis C virus infection and alcohol exposure. Hepatology.

[25] Fan AC (2009). Nanofluidic proteomic assay for serial analysis of oncoprotein activation in clinical specimens. Nat. Med..

[26] Cargnello M, Roux PP (2011). Activation and function of the MAPKs and their substrates, the MAPK-activated protein kinases. Microbiol. Mol. Biol. Rev..

[27] Steelman LS (2011). Roles of the Raf/MEK/ERK and PI3K/PTEN/Akt/mTOR pathways in controlling growth and sensitivity to therapy-implications for cancer and aging. Aging.

[28] Zhao Y, Adjei AA (2014). The clinical development of MEK inhibitors. Nat. Rev. Clin. Oncol..

[29] Roberts PJ, Der CJ (2007). Targeting the Raf-MEK-ERK mitogen-activated protein kinase cascade for the treatment of cancer. Oncogene.

[30] Caunt CJ, Sale MJ, Smith PD, Cook SJ (2015). MEK1 and MEK2 inhibitors and cancer therapy: the long and winding road. Nat. Rev. Cancer.

[31] O’Neill RA (2006). Isoelectric focusing technology quantifies protein signaling in 25 cells. Proc. Natl. Acad. Sci. USA.

[32] Johlfs MG, Gorjala P, Urasaki Y, Le TT, Fiscus RR (2015). Capillary isoelectric focusing immunoassay for fat cell differentiation proteomics. PLoS One.

[33] Moy B, Kirkpatrick P, Kar S, Goss P (2007). Lapatinib. Nat. Rev. Drug Discov..

[34] Holderfield M, Deuker MM, McCormick F, McMahon M (2014). Targeting RAF kinases for cancer therapy: BRAF-mutated melanoma and beyond. Nat. Rev. Cancer.

[35] Samatar AA, Poulikakos PI (2014). Targeting RAS-ERK signalling in cancer: promises and challenges. Nat. Rev. Drug Discov..

[36] Paruch K (2010). Discovery of dinaciclib (SCH 727965): a potent and selective inhibitor of cyclin-dependent kinases. ACS Med. Chem. Lett..

[37] Liu X (2015). Targeting polo-like kinases: a promising therapeutic approach for cancer treatment. Transl. Oncol..

[38] Sharma P (2002). Phosphorylation of MEK1 by cdk5/p35 down-regulates the mitogen-activated protein kinase pathway. J. Biol. Chem..

[39] Harding A, Giles N, Burgess A, Hancock JF, Gabrielli BG (2003). Mechanism of mitosis-specific activation of MEK1. J. Biol. Chem..

[40] Zheng CF, Guan KL (1994). Activation of MEK family kinases requires phosphorylation of two conserved Ser/Thr residues. EMBO J..

[41] Eblen ST (2004). Mitogen-activated protein kinase feedback phosphorylation regulates MEK1 complex formation and activation during cellular adhesion. Mol. Cell. Biol..

[42] Tassin TC, Benavides DR, Plattner F, Nishi A, Bibb JA (2015). Regulation of ERK kinase by MEK1 kinase inhibition in the brain. J. Biol. Chem..

[43] Brunet A, Pages G, Pouyssegur J (1994). Growth factor-stimulated MAP kinase induces rapid retrophosphorylation and inhibition of MAP kinase kinase (MEK1). FEBS Lett..

[44] Mansour SJ (1994). Mitogen-activated protein (MAP) kinase phosphorylation of MAP kinase kinase: determination of phosphorylation sites by mass spectrometry and site-directed mutagenesis. J. Biochem..

[45] Chen JQ, Wakefield LM, Goldstein DJ (2015). Capillary nano-immunoassays: advancing quantitative proteomics analysis, biomarker assessment, and molecular diagnostics. J. Transl. Med..

[46] Aspinall-O’Dea M (2015). Antibody-based detection of protein phosphorylation status to track the efficacy of novel therapies using nanogram protein quantities from stem cells and cell lines. Nat Prot..

[47] Urasaki Y, Pizzorno G, Le TT (2016). Chronic uridine administration induces fatty liver and pre-diabetic conditions in mice. PLoS One.

[48] Schrotter S, Leondaritis G, Eickholt BJ (2016). Capillary isoelectric focusing of Akt isoforms identifies highly dynamic phosphorylation in neuronal cells and brain tissue. J. Biol. Chem..

[49] Iacovides DC (2013). Identification and quantification of AKT isoforms and phosphoforms in breast cancer using a novel nanofluidic immunoassay. Mol. Cell. Prot..

[50] Guo H (2014). Coordinate phosphorylation of multiple residues on single AKT1 and AKT2 molecules. Oncogene.

[51] Dai J, Lamp J, Xia Q, Zhang Y (2018). Capillary isoelectric focusing-mass spectrometry method for the separation and online characterization of intact monoclonal antibody charge variants. Anal. Chem..

[52] McCubrey JA (2007). Roles of the Raf/MEK/ERK pathway in cell growth, malignant transformation and drug resistance. Biochim. Biophys. Acta.

[53] Shaul YD, Seger R (2007). The MEK/ERK cascade: from signaling specificity to diverse functions. Biochim. Biophys. Acta.

[54] Jokinen E, Koivunen JP (2015). MEK and PI3K inhibition in solid tumors: rationale and evidence to date. Ther. Adv. Med. Oncol..

[55] Wei W (2016). Single-cell phosphoproteomics resolves adaptive signaling dynamics and informs targeted combination therapy in glioblastoma. Cancer Cell.

[56] Lawrence RT (2015). The proteomic landscape of triple-negative breast cancer. Cell Rep..

[57] Irish JM (2004). Single cell profiling of potentiated phospho-protein networks in cancer cells. Cell.

[58] Neve RM (2006). A collection of breast cancer cell lines for the study of functionally distinct cancer subtypes. Cancer Cell.

[59] Jacquemier J (2005). Protein expression profiling identifies subclasses of breast cancer and predicts prognosis. Cancer Res..

