# Supplementary material for: Detection of the Cell Cycle-Regulated Negative Feedback Phosphorylation of Mitogen-Activated Protein Kinases in Breast Carcinoma using Nanofluidic Proteomics
Source: Sci Rep. 2018 Jul 3;8:9991. doi: 10.1038/s41598-018-28335-8 (PMC6030070; doi:10.1038/s41598-018-28335-8)
Supplement: Supplementary file 1 — Supplementary Information [file 41598_2018_28335_MOESM1_ESM.pdf]

Supplementary Information for

Detection of the Cell Cycle-Regulated Negative Feedback Phosphorylation of Mitogen-Activated Protein Kinases in Breast Carcinoma using Nanofluidic Proteomics

Yasuyo Urasaki[1], Ronald R. Fiscus[2], Thuc T. Le[1]*

[1]College of Pharmacy & [2]College of Medicine, Roseman University of Health Sciences, 10530 Discovery Drive, Las Vegas, NV 89135, USA

*Correspondence and requests for materials should be addressed to T.T.L. (email: tle5@roseman.edu)

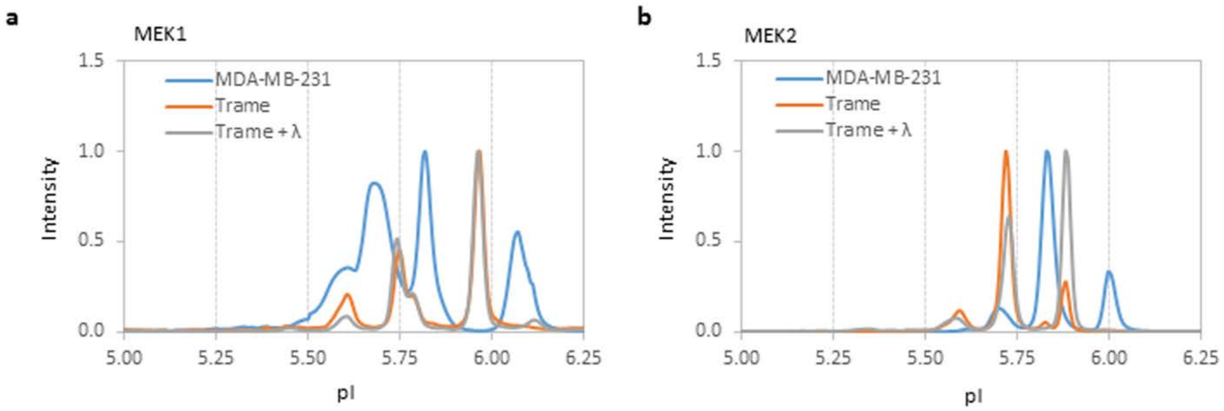

Supplemental Figure S1. Trametinib treatment caused shifts toward lower pI values for (a) MEK1 and (b) MEK2 that were resistant to λ phosphatase treatment. Blue line: MDA-MB-231; orange line: MDA-MB-231 cells treated with trametinib; grey line: MDA-MB-231 cells treated with trametinib and TCEs were subsequently treated with λ phosphatase.

Supplemental Table S1. List of primary antibodies used for Western blots and cIEF immunoassays

Antibody	Cat. No.	Vendor
MEK1	07-641	Millipore (Billerica, MA)
pMEK1 (Ser217/Ser221)	9121	Cell Signaling (Danvers, MA)
pMEK1 (Thr286)	9127	Cell Signaling (Danvers, MA)
pMEK1 (Thr292)	26975	Cell Signaling (Danvers, MA)
pMEK1 (Ser298)	9128	Cell Signaling (Danvers, MA)
pMEK1 (Thr386)	180-386	PhosphoSolutions (Aurora, CO)
MEK2	9125	Cell Signaling (Danvers, MA)
pMEK2 (Thr394)	30622	Abcam (Cambridge. MA)
ERK1/2	040-474	Protein Simple (Santa Clara, CA)
p-ERK1/2 (Thr202/Tyr204)	040-477	Protein Simple (Santa Clara, CA)
ERK1	05-957	Millipore (Billerica, MA)
ERK2	06-333	Millipore (Billerica, MA)
